# Supplementary material for: Application of Exogenous Melatonin Improves Tomato Fruit Quality by Promoting the Accumulation of Primary and Secondary Metabolites
Source: Foods. 2022 Dec 19;11(24):4097. doi: 10.3390/foods11244097 (PMC9778358; doi:10.3390/foods11244097)
Supplement: Supplementary file 1 [file foods-11-04097-s001.zip › foods-2099337-supplementary.pdf]

Supplementary Table S1. Contents of polyphenolic components in tomato fruit at 52 d after anthesis (μg·g^{-1} DW).

polyphenolic components		CK-1	CK-2	CK-3	T1-1	T1-2	T1-3	T2-1	T2-2	T2-3
Flavonoids	Rutin	556.5754	594.2606	578.0122	648.4106	690.4766	640.3847	676.3897	766.3663	681.5402
	Quercetin	787.3116	741.9344	671.9594	725.5894	826.0883	829.6275	963.1528	872.6549	864.0458
	Naringenin	5.8160	4.3456	5.7556	9.4355	9.5139	8.3205	12.2060	12.8980	12.9992
	Kaempferol	2.7021	1.8556	1.5909	2.4919	4.3873	4.3706	2.2527	3.2983	3.2561
Phenolic acids	Protocatechuic acid	75.7707	78.0208	75.2022	88.5280	75.7945	86.7358	83.9446	89.3819	94.3317
	P-hydroxybenzoic acid	3.0488	4.9585	4.4809	3.6540	4.2234	3.7701	6.5629	4.7563	6.0430
	Chlorogenic acid	258.8587	255.1315	280.7831	265.8248	295.7651	268.2954	343.5162	309.7163	351.6171
	Gallic acid	35.7728	33.6631	36.6943	43.1021	52.7940	44.9126	39.9995	36.6576	47.7969
	4-Coumaric acid	3.1814	2.6196	2.8304	2.7051	2.2084	2.7126	3.5535	2.5985	2.5904
	Ferulic acid	2.0939	3.5946	2.7071	3.6030	2.7364	2.9212	3.0701	5.0435	2.3173
	Benzoic acid	63.8522	88.8934	108.2008	98.4786	78.6029	89.8626	105.1766	85.5343	91.8466
	Cinnamic acid	0.7754	1.1933	1.2112	2.2840	2.3073	1.9536	2.0222	1.4498	1.5675
	Gentisic acid	12.1747	13.8268	13.6631	12.6046	14.9355	13.0859	21.0235	17.4890	15.8532
	Caffeic acid	7.0114	6.4515	7.7776	6.6069	7.5738	8.8883	8.3575	8.9515	10.7136
	Cynarin	0.8286	0.4572	0.5411	1.5514	1.3163	1.8746	1.3313	1.8854	2.0275
	Sinapic acid	0.9287	0.6736	0.7116	1.4097	1.7851	1.2497	2.1234	1.4632	1.6940

Supplementary Table S2. Volatile flavor intensity in tomato fruit at 52 d after anthesis.

Sensors	CK-1	CK-2	CK-3	T1-1	T1-2	T1-3	T2-1	T2-2	T2-3
W1C	0.1664	0.1668	0.1671	0.1853	0.1859	0.1864	0.2148	0.2149	0.2152
W5S	11.2401	11.9247	11.6457	13.1752	14.2157	13.2801	15.8122	14.4105	14.3575
W3C	0.3539	0.3410	0.3522	0.3581	0.3425	0.3442	0.3546	0.3657	0.3603
W6S	1.1099	1.1106	1.1108	1.1212	1.1250	1.1186	1.1192	1.1325	1.1210
W5C	0.5756	0.5762	0.5738	0.5967	0.5809	0.5836	0.6025	0.6088	0.6069
W1S	1.0116	1.0193	1.0021	1.0147	1.0153	1.0131	1.0149	1.0140	1.0186
W1W	1.0106	1.0124	1.0010	1.0147	1.0164	1.0202	1.0168	1.0221	1.0131
W2S	1.0113	1.0095	1.0057	1.0134	1.0161	1.0104	1.0240	1.0174	1.0162
W2W	3.7732	3.6629	3.8673	4.2118	4.1979	4.1828	4.5937	4.5684	4.5551
W3S	1.9011	1.9012	1.9186	1.8621	1.8448	1.8611	1.8766	1.7791	1.8339
